# WHO declares the end of the COVID-19 global health emergency: lessons and recommendations from the perspective of ChatGPT/GPT-4

**DOI:** 10.1097/JS9.0000000000000521

**Published:** 2023-05-26

**Authors:** Kunming Cheng, Chunchun Wu, Shuqin Gu, Yanqiu Lu, Haiyang Wu, Cheng Li

**Affiliations:** a Department of Intensive Care Unit, The Second Affiliated Hospital of Zhengzhou University, Zhengzhou; b Department of Emergency, Taikang People’s Hospital, Zhoukou, Henan; c Department of Orthopaedic Surgery, Beijing Jishuitan Hospital, Fourth Clinical College of Peking University; d State Key Laboratory of Toxicology and Medical Countermeasures, Beijing Institute of Pharmacology and Toxicology, Beijing; e Clinical College of Neurology, Neurosurgery and Neurorehabilitation; f Department of Graduate School, Tianjin Medical University, Tianjin, People’s Republic of China; g Duke Human Vaccine Institute, Duke University Medical Center; h Duke Molecular Physiology Institute, Duke University School of Medicine, Durham, North Carolina, USA; i Center for Musculoskeletal Surgery (CMSC), Charité-Universitätsmedizin Berlin, corporate member of Freie Universität Berlin, Humboldt University of Berlin, and Berlin Institute of Health, Berlin, Germany

*Dear Editor*,

On 5 May 2023, the World Health Organization (WHO) announced that the coronavirus disease 2019 (COVID-19) epidemic would no longer be listed as a public health emergency of international concern (PHEIC), 3 years after the first PHEIC alert on 30 January 2020^1^. The WHO committee highlighted the reduction in death rates and hospitalizations, along with the increased levels of population immunity against severe acute respiratory syndrome coronavirus 2 (SARS-CoV-2), as reasons for ending the PHEIC status. To some extent, this could be regarded as a symbol of the end of the global COVID-19 pandemic and we are witnessing a turning point of the most severe emergent infectious disease pandemic in the 21st century^2^.

Moreover, as we know that artificial intelligence (AI) technology includes machine learning, deep learning, data analysis, and data mining, is an important scientific approach to studying the spread routes, spread processes, and epidemic laws of infectious diseases and has played a critical role in effective prevention, control, and elimination of the global COVID-19 pandemic. Based on the lessons learned from the COVID-19 pandemic, some scholars believe that AI-enabled clinical trials might be a faster way to conduct rapid clinical trials and counter future pandemics^3^. In addition, when referring to AI, an exponentially growing AI application called ChatGPT, a general large language model (LLM) developed by OpenAI company, has attracted substantial interest from researchers. GPT refers to Generative Pre-training Transformer and GPT-4 is the latest version of OpenAI’s LLM system^4^. Recently, numerous studies have explored the potential of ChatGPT/GPT-4 in the biomedical field, including medical imaging, virtual assistant, clinical decision-making support, health management, precision medicine, and so on. As for the field of infectious diseases, our research group has analyzed the most concerned questions about the use of ChatGPT in the infectious disease domain^5,6^. Additionally, several scholars also acquired answers and recommendations from ChatGPT on the frequently asked questions about COVID-19, such as spread, symptoms, diagnosis, treatment, vaccines, and pandemic management^7^. At the time point of WHO announced the end of COVID-19 PHEIC, we conducted an online survey using GPT-4 (https://chat.openai.com/chat).

## Continued vigilance is needed

PHEIC represents the highest level of alert issued by WHO under the International Health Regulations (IHR). It refers to an unusual event that poses a public health risk to other countries through the international spread of disease, warranting a coordinated international response. Since 2009, the WHO has declared seven international public health emergencies, including the H1N1 influenza pandemic, polio outbreak, Ebola outbreak in West Africa, Zika epidemic, Ebola outbreak in Congo, COVID-19, and Mpox (monkeypox), with the first being the H1N1 pandemic in Mexico and the United States. On 30 January 2020, in response to the escalating global threat, the WHO declared the COVID-19 pandemic as a PHEIC. As of now, the cumulative number of confirmed COVID-19 cases worldwide has reached 760 million, with 6.92 million cumulative deaths. Approximately 5 billion people have received at least one dose of vaccine. Of note, COVID-19 is no longer deemed a PHEIC does not imply the virus or disease has vanished, but rather that we have the ability to effectively control the epidemic. With this downgrade, countries/regions are no longer required to implement mandatory public health measures and control strategies. This shift is expected to stimulate new developments across the global and national medical care, transportation, trade, and other sectors^1,2^. Nevertheless, many experts maintain that we are only at the beginning of the journey toward long-term COVID-19 management. As illustrated in Supplementary Figure 1 (Supplemental Digital Content 1, http://links.lww.com/JS9/A636), GPT-4 also believe that the WHO’s decision to no longer classify COVID-19 as a PHEIC does not necessarily mean that the disease is no longer a health threat. Continuous research indicates that COVID-19 continues to mutate, and sporadic outbreaks persist worldwide, suggesting the disease still poses a risk. For instance, one study from China CDC Weekly showed that SARS-CoV-2 reinfections were monitored in Guangdong Province between December 2022 and January 2023^8^. According to their estimates, the reinfection incidence was 50.0% for the original strain primary infections, 35.2% for the Alpha or Delta variants, and 18.4% for the Omicron variant. Up to now, the Chinese government continues to manage COVID-19 using measures applicable to Class B infectious diseases. Therefore, in alignment with GPT-4, we believe that ongoing vigilance, surveillance, testing, and localized responses to potential outbreaks remain necessary. Furthermore, the established prevention and monitoring mechanisms for COVID-19, including surveillance, contact tracing, and testing, may still be required in the future.

## Long-term response plan

**Figure 1:**
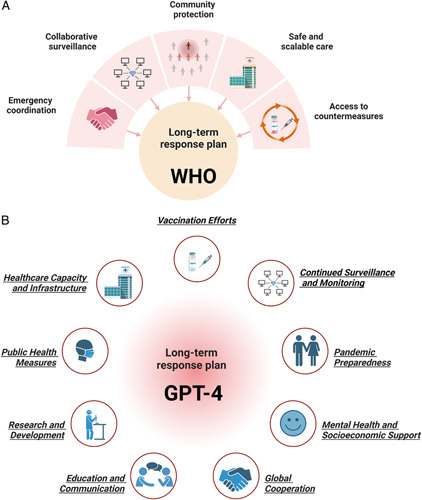
Long-term response plan from WHO (A) and GPT-4 (B).

While recognizing the ongoing uncertainties posed by the potential evolution of SARS-CoV-2, experts from IHR emergency committee suggest that it is time to transition toward the long-term management of the COVID-19 pandemic. To address this challenge, the WHO released a strategic plan on 3 May 2023, outlining the transition from emergency status to long-term disease management of COVID-19 for the period of 2023–2025. The plan presents 10 essential recommendations organized into 5 primary areas: emergency coordination, collaborative surveillance, community protection, safe and scalable care, and access to countermeasures (Fig. 1A). These guidelines will assist countries in their crucial roles within the long-term management of COVID-19 while safeguarding public health and safety. In addition, as shown in Figure 1B and Supplementary Figure 2 (Supplemental Digital Content 2, http://links.lww.com/JS9/A637), GPT-4 also gives nine recommendations for COVID-19 long-term management. These recommendations concern continued surveillance and monitoring, vaccination efforts, healthcare capacity and infrastructure, public health measures, research and development, education and communication, global cooperation, mental health and socioeconomic support, as well as pandemic preparedness. It is not difficult to see that GPT-4 seems to give a more comprehensive recommendation checklist. Combining the suggestions from WHO and GPT-4, long-term COVID-19 surveillance and vaccine development are still the top priorities. Take vaccination as an example; under the circumstances that current vaccines and antiviral drugs are not capable of completely ending the COVID-19 epidemic, the future market still needs an ideal COVID-19 vaccine that is broad-spectrum efficient and could induce long-term immune protection. And if new strains of the COVID-19 virus emerge that demonstrate resistance to current vaccines, it may be necessary to develop and distribute updated vaccines. Additionally, the COVID-19 vaccine also has a high possibility of becoming part of routine healthcare, similar to the annual flu shot.

## Lessons for surgeons

The unprecedented scale and reach of the COVID-19 pandemic have compelled healthcare systems, already under significant strain, to undergo adaptive changes and evolution. As a result, there has been a considerable shift in practices, altering the roles and expectations of many physicians. In the domain of surgery, the pandemic has induced a paradigm shift, leading to an ongoing evolution of clinical guidelines. Surgeons have found themselves reassessing nearly every facet of their daily clinical practices. This reevaluation has included the deferral of non-emergent surgical procedures. Elective surgeries have been canceled, and the provision of clinic services has been curtailed. Therefore, at this historical turning point, it is of great importance to collate the experiences and lessons learned by surgeons during the COVID-19 pandemic. Such a compilation will aid surgeons in accumulating knowledge and will better equip them to tackle potential future outbreaks of infectious diseases. As shown in Supplementary Figure 3 (Supplemental Digital Content 3, http://links.lww.com/JS9/A638) GPT-4 has briefly summarized seven key points for surgeons, including elective surgery and prioritization, telemedicine, personal protective equipment (PPE) and infection control, preoperative testing and patient management, well-being and mental health, collaboration and flexibility, and research and innovation. Take well-being and mental health as examples; in fact, the change from the COVID-19 pandemic and work overload has generated significant stress and strain on healthcare professionals and surgeons. It is reported that the mental health of healthcare professionals has worsened since the pandemic’s second year^9^. Numerous previous studies also have found that 50% of all surgeons experienced at least one COVID-19-related burnout^10^. Moreover, with the start of a long-term response plan, the global fight against the COVID-19 virus appears to be a long battle. Although COVID-19 global health emergency was finally declared over, the psychological impact on clinicians may linger long. It is clear, therefore, that larger studies are needed to gain a more comprehensive understanding of the needs of surgeons in the current situation. Several studies have noticed this phenomenon and called for strategies to address pandemic-related burnout.

## Conclusion

All in all, the WHO announced that the COVID-19 epidemic was no longer listed as a global public health emergency, which showed that we have made important progress in controlling this epidemic. However, the end of PHEIC does not mean COVID-19 is no longer a health threat. In the future, it may co-exist with humans for a long time, and there is a need for constant vigilance. Meanwhile, it is time to transition toward the long-term management of the COVID-19 pandemic. Continuous surveillance and vaccine development are still the top priorities. In addition, although the war against the epidemic has not been declared a final victory, at this historic moment, we should express gratitude for the efforts made by people all around the world. Let us all work together to continue to fight COVID-19.

## Ethical approval

This study does not include any individual-level data and thus does not require any ethical approval.

## Sources of funding

This study is supported by China Postdoctoral Science Foundation (2022M720385) and Beijing JST Research Funding (YGQ-202313).

## Author contribution

K.C.: conceptualization, methodology, data curation, formal analysis, investigation, and writing – original draft; C.W.: conceptualization, methodology, data curation, and formal analysis; S.G.: data curation, formal analysis, resources, and investigation; Y.L.: data curation, formal analysis, resources, and investigation; H.W. conceptualization, methodology, data curation, formal analysis, resources, and investigation; C.L.: conceptualization, methodology, data curation, and formal analysis.

## Conflicts of interest disclosure

The authors declare no conflicts of interest.

## Research registration unique identifying number (UIN)

1. Name of the registry: not applicable.
2. Unique identifying number or registration ID: not applicable.
3. Hyperlink to your specific registration (must be publicly accessible and will be checked): not applicable.

## Guarantor

Haiyang Wu and Cheng Li.

## Data availability statement

The data underlying this article will be shared by the corresponding author upon reasonable request.

## Supplementary Material

SUPPLEMENTARY MATERIAL
