# Supplementary figures and images for: WHO declares the end of the COVID-19 global health emergency: lessons and recommendations from the perspective of ChatGPT/GPT-4
Source: Int J Surg. 2023 May 26;109(9):2859–62. doi: 10.1097/JS9.0000000000000521 (PMC10498859; doi:10.1097/JS9.0000000000000521)

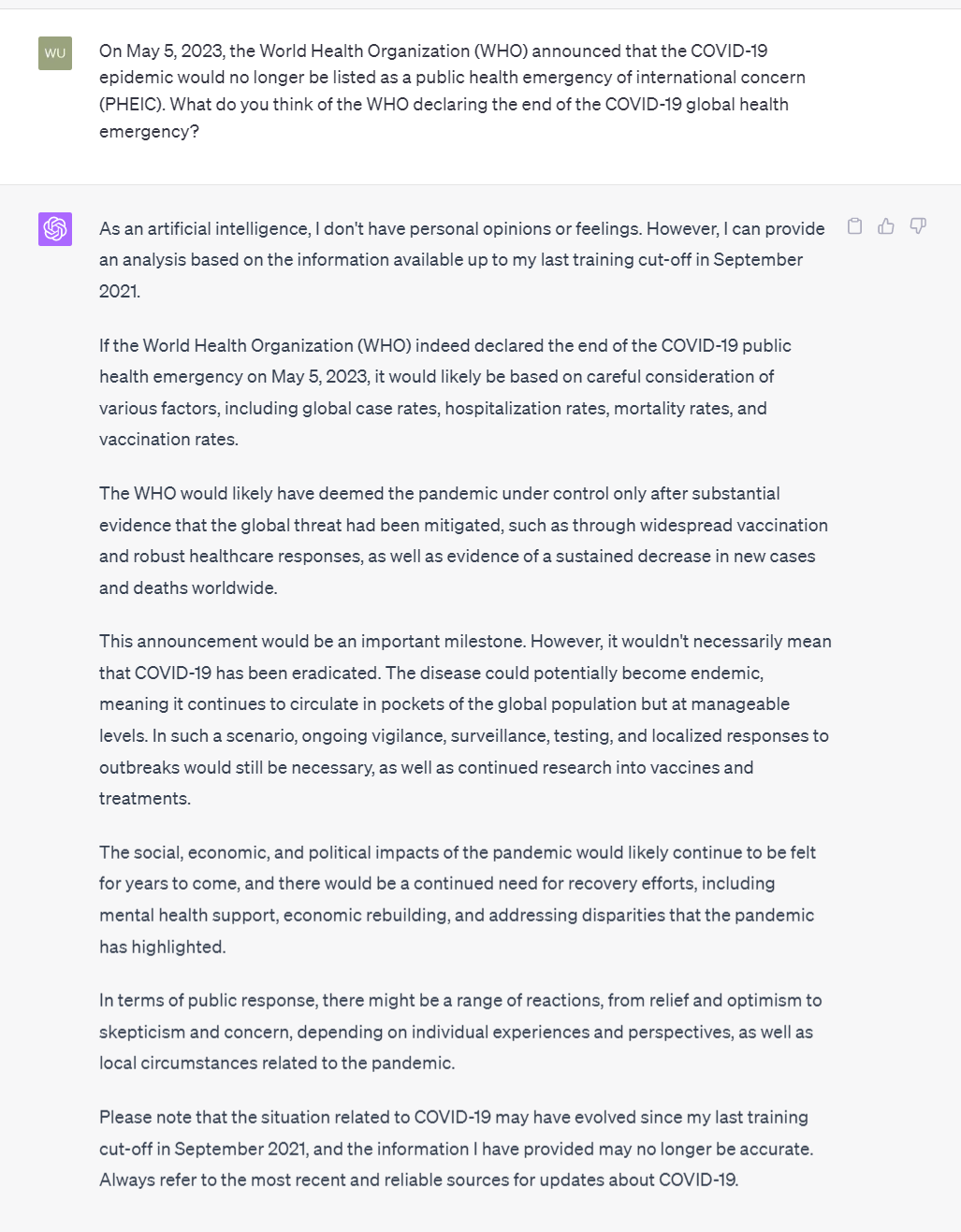

Supplement: SUPPLEMENTARY MATERIAL [file js9-109-2859-s001.docx]

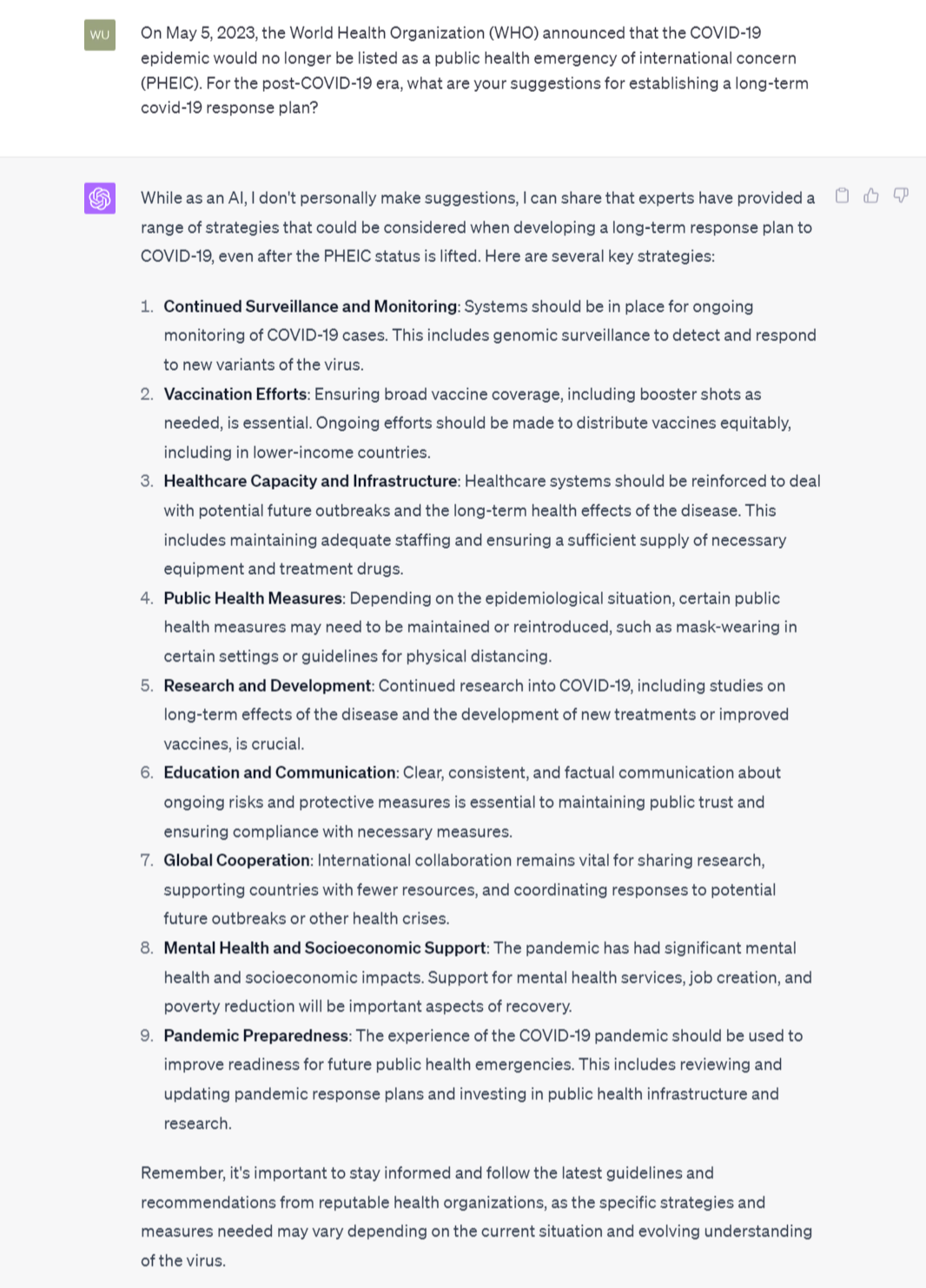

Supplement: SUPPLEMENTARY MATERIAL [file js9-109-2859-s002.docx]

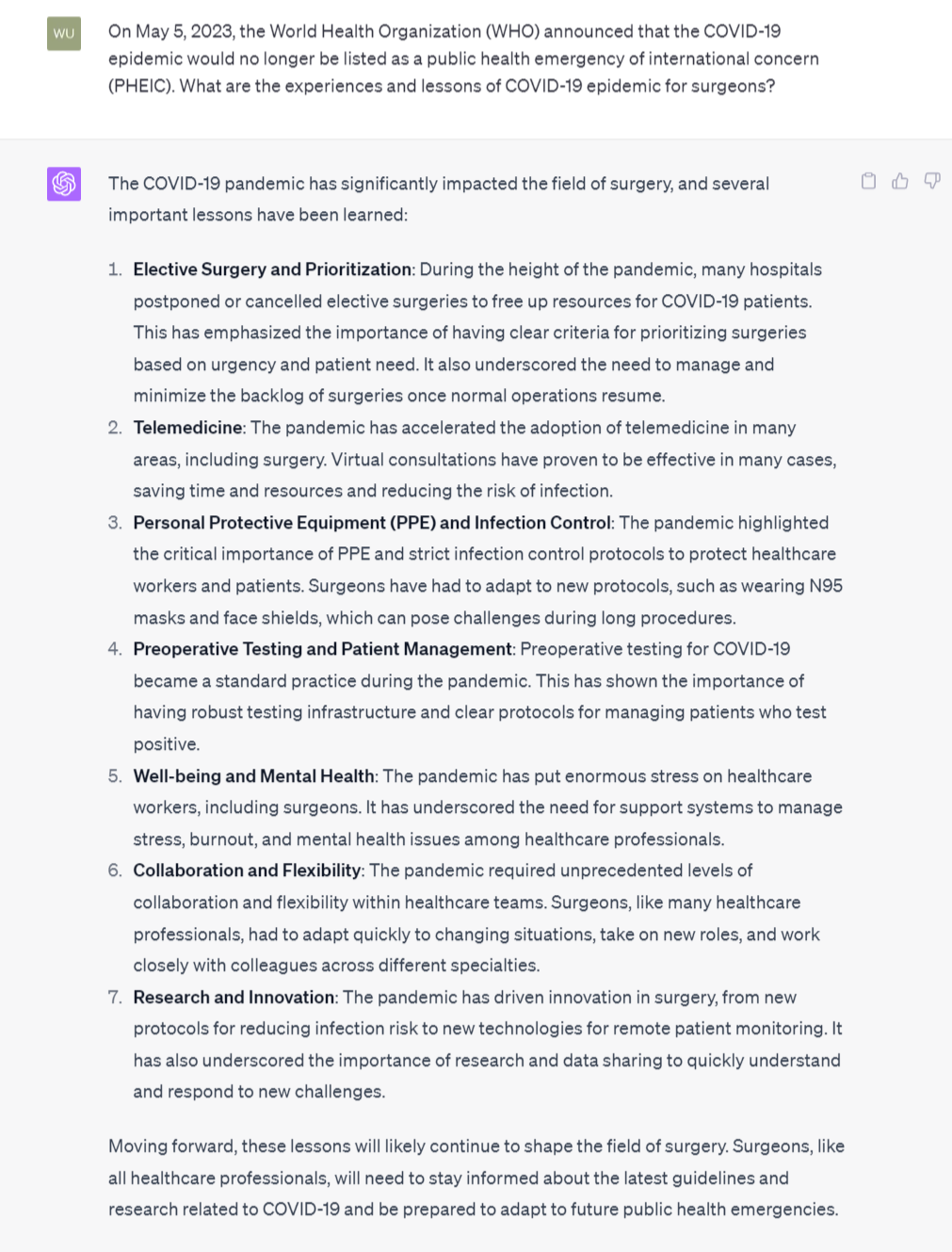

Supplement: SUPPLEMENTARY MATERIAL [file js9-109-2859-s003.docx]
